# Cold Plasma Treatment of Quinoa Grains: Changes in Phytic Acid, Saponin, Content, and Antioxidant Capacity

**DOI:** 10.1002/fsn3.4691

**Published:** 2024-12-19

**Authors:** Sanaz Arjmand, Elham Khalili Sadrabad, Fereshteh Ramroudi, Neda Mollakhalili‐meybodi

**Affiliations:** ^1^ Research Center for Food Hygiene and Safety, School of Public Health Shahid Sadoughi University of Medical Sciences Yazd Iran; ^2^ Department of Food Hygiene and Safety, School of Public Health Shahid Sadoughi University of Medical Sciences Yazd Iran; ^3^ Department of Food Science and Technology, School of Public Health Shahid Sadoughi University of Medical Sciences Yazd Iran

**Keywords:** antioxidant capacity, cold plasma, quinoa, saponin and phytic acid

## Abstract

The impact of atmospheric cold plasma (ACP) treatment (at 50 and 60 kV for 5 and 10 min) on nutritional (total phenolic and flavonoids contents, antioxidant capacity, and TBARs) and antinutritional (saponin and phytic acid) characteristics of quinoa grains has been investigated at this study. Results indicated that ACP treatment is significantly effective to reduce the antinutritional compounds compared with the control sample (*p* ≤ 0.05), among which S_4_ (i.e., treated at 60 kV for 10 min) and S_2_ (i.e., treated at 50 kV for 10 min) samples showed the highest decrease in saponin and phytic acid content, respectively. Also, total phenolic content and antioxidant capacity (DPPH and FRAP) of ACP‐treated samples have decreased compared with the control sample. The flavonoid content of ACP‐treated samples has been increased compared with the control sample (*p* ≤ 0.05). In general, the S_4_ (at 60 kV for 10 min) samples had the highest amount of flavonoid and phenolic content compared with the other samples. A significant reduction in TBAR values has been observed by ACP treatment with the maximum reduction at S_4_ (i.e., treated at 60 kV for 10 min) samples. Results indicated that ACP treatment at 60 KV for 10 min is effective to reduce the antinutritional compounds and maintain the antioxidant compounds of quinoa grains as well. Considering the necessity of keeping the nutritional characteristics of grains through processing, it needs to be monitored and optimized the condition in a way that nutritional characteristics are preserved.

## Graphical abstract

According to the results, by considering the reduction in antinutritional compounds reduction as well as the preservative effects on the antioxidant properties of quinoa grains, the cold plasma treatment at 60 kV for 10 min was recommended.

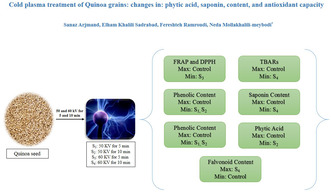

## 1 Introduction

Quinoa is a gluten‐free pseudo‐grain, with high quality and quantity of protein with high amounts of the amino acid lysine and sulfur amino acids, containing all essential amino acids (Vilcacundo and Hernández‐Ledesma 2017; Zare et al. 2022), significant content of vitamins, dietary fiber, and minerals (Zare et al. 2022). Whole grain quinoa contains high quantity of antioxidants such as polyphenolic compounds, carotenoids, vitamin C, and flavonoids that play a beneficial role in protecting against diseases including cancer, allergies, and inflammation which reduce the risk of cardiovascular disease (Dini, Tenore, and Dini 2010; Yael et al. 2012). Vitamin E (tocopherols) is another major ingredient in quinoa seeds with antioxidant properties that stabilize chloroplast membranes by reaction with polyunsaturated acyl groups of lipids, so reduce different reactive oxygen species and by‐products of oxidative stress (Miranda et al. 2010).

Saponin is a broad group of water‐soluble glycosides triterpenoids and steroids found in various parts of the quinoa including seeds, seed coats, flowers, and fruits (James 2009; Lim, Park, and Yoon 2020). Saponin is the main antinutritional compound in quinoa seed coatings, which causes a bitter taste in samples with a value of more than 1.1 mg/g (Koziol 1991; Suárez‐Estrella et al. 2018). So far, several studies have been conducted as effective treatments to reduce and eliminate saponin in quinoa, such as immersion, grain washing, boiling, roasting, extrusion, and heat utilization. Also, genetic methods have been used to produce quinoa with less saponin (El Hazzam et al. 2020). Due to the adverse effects of these treatments such as reducing phenolic compounds, vitamins and minerals, changes in grain structure, water pollution, additional cost of drying seeds, and increasing the possibility of seed germination, low efficiency, and high time, the attention have been paid to use alternative treatments to reduce quinoa bitterness by breaking glycosidic bonds (Suárez‐Estrella et al. 2018; El Hazzam et al. 2020; Wie et al. 2007).

In recent decades, food safety has emerged as a public health issue worldwide with economic and political implications. Antinutritional agents are one of the primary concerns associated with food products due to their potential adverse health effects. Despite the efficiency of various conventional processing methods, including thermal processing methods (such as boiling, frying, roasting, and baking) to reduce or eliminate antinutritional factors from food, they may lead to a significant reduction in nutrients (Xiang et al. 2023). Nonthermal processing methods, including high pressure processing, pulsed electric fields, and cold plasma treatment are emerging as promising alternatives to preserve nutritional value while ensuring food safety. In this regard, atmospheric cold plasma (ACP) treatment is recognized as green processing which is done at low pressure has shown great potential for reducing food hazards (Gupta et al. n.d.).

Plasma, the fourth state of matter, contains a balanced combination of free electrons, photons, and neutral atoms and is electrically neutral due to the equality of its positive and negative ions (Zare et al. 2022). Plasma treatment has the ability to affect various bonds, including glycosidic bonds (Jeong et al. 2004; Patras et al. 2009; Golmohamadi et al. 2013). ACP is a physical technique of food processing which has been investigated due to its characteristics such as the presence of active species, low temperature, and no need to vacuum in the food industry (Baier et al. 2013; Misra et al. 2015).

During ACP treatment, reactive species interact with macromolecules and encourage the cross‐linking, depolymerization, surface etching, oxidation, and hydroxylation. Interestingly, ACP treatment was also used to inactivate the enzymes (e.g., polyphenol oxidase, peroxidase, pectin, and lipoxygenase). Similarly, ACP treatment was applied to improve the functional, morphological, and rheological properties of jackfruit seed flour, red Adzuki bean starch, wheat flour, pearl millet, little millet, black gram, tapioca starch, and quinoa (Kheto et al. 2023).

Considering the potential role of ACP treatment in breakage of the glycosidic bonds as an effective way in reducing the bitterness of saponin (Han, Shi, and Sun 2020; Zahoranová et al. 2018), this study aimed to evaluate the efficiency of cold plasma treatment as one of the effective treatments in reducing saponin content. Also, several studies show the adverse effect of various treatments such as heating processes (Jeong et al. 2004), high hydrostatic pressure (Keenan et al. 2010), ultrasound treatment (Golmohamadi et al. 2013), freezing, and cooking operations (Danesi and Bordoni 2008) on the amount and activity of antioxidants in different foods. Therefore, total flavonoid content (TFC), the total phenolic content (TPC), and antioxidant activity (DPPH and FRAP) of treated samples were also investigated.

## 2 Materials and Methods

### 2.1 Materials

Quinoa grains (*Titicaca*) were prepared from a local market in Yazd, Iran. All chemicals and reagents used were of analytical grade.

### 2.2 Sample Preparation

Quinoa grains exposed to plasma treatment at 50 and 60 kV for 5 and 10 min with four groups including S_1_: the plasma‐treated samples at 50 KV for 5 min, S_2_: the plasma‐treated samples at 50 KV for 10 min, S_3_: the plasma‐treated samples at 60 KV for 5 min and S_4_: the plasma‐treated samples at 60 KV for 10 min. Control sample had not exposed to any plasma treatment. Then, whole quinoa grains were grinded and were passed through No. 50 mesh sieve. Flour samples were gathered for further analysis.

### 2.3 Antioxidant Extraction

Methanol extraction of samples were prepared according to method of Amiri et al., by adding 20 mL methanol to 1 g of flour samples and shaking for 2 h. The contents were centrifuged at 2500 × *g* for 10 min, and the extraction was done in triplicate for sediments. Then, the supernatant were collected and kept at −20°C for antioxidant activity analysis.

#### 2.3.1 Total Phenolic Content

Total phenolic content was measured according to Ghasemi et al. method (Ghasemi et al. 2023) using Folin–Ciocalteu's reagent (FCR). About, 2.5 mL Folin–Ciocalteu's reagent (10 times diluted) was added to 500 μL of extracted sample, after 6 min Na_2_CO_3_ 7.5% (2 mL) was added. The samples were kept at dark room temperature for 60 min. The absorbance of the samples was recorded at 765 nm. The total amount of phenolic content was expressed as mg of Gallic acid equivalent per 100 g dry sample (mg GAE/100 g).

#### 2.3.2 Total Flavonoid Content

The mixture of extracted samples (250 μL), deionized distilled water (1.25 mL), and sodium nitrite (75 μL, 5% v/w) remained for 6 min at room temperature. Thereafter, aluminum chloride hydrate (150 μL) was added and remained for 5 min. Finally, 275 μL of ethanol and 0.5 mL sodium hydroxide solution (1 M) were added and the absorbance of the samples was read at 510 nm. The total amount of flavonoid content was expressed as mg of rutin equivalent per 100 g dry sample (mg rutin/100 g) (Amiri et al. 2023).

#### 2.3.3 DPPH Assay

To evaluate DPPH, 0.5 mL extracted sample was mixed with 2.5 mL 2, 2‐diphenyl‐1‐ picrylhydrazyl (DPPH). The absorbance of samples was recorded after 30 min at 517 nm against methanol (Saeid et al. 2023). The radical scavenging activity of DPPH was calculated as follows.

$$\left(\%\right)\;\text{radical scavenging activity}:\left[\left(A_{\text{control}}–A_{\text{sample}}\right)/A_{\text{control}}\right]\times 100$$

Where the A_control_ and A_sample_ determined is amount of control absorption and sample absorption, respectively.

#### 2.3.4 FRAP Assay

FRAP was measured according to Ghasemi et al. method (Ghasemi et al. 2023) in which 500 μL of extracted samples was mixed with 3 mL of FRAP reagent (2,4,6‐tripyridyl‐s‐triazine, FeCl_3_∙6H_2_O, and acetate buffer in ratio of 1:1:10). The absorbance of samples was recorded at 593 nm, and the results were expressed as mmol Fe_2_SO_4_.

### 2.4 TBARs Assay

About, 0.5 g of the sample was poured into 10 mL of 0.1% trichloroacetic acid (TCA) solution and centrifuged at 8500 rpm for 5 min. Then, the supernatant solution (2 mL) was added to TBA (4 mL, thiobarbituric acid 0.5% + 20% TCA solution). The samples were kept at 95°C for 30 min. Then the samples were cooled and centrifuged at 8500 rpm for 10 min. The absorbance of the samples was read at 532 nm against distilled water as a blank. The TBARs was expressed as mg of malondialdehyde/kg dry sample (Ghasemi et al. 2023).

### 2.5 Saponin Content

Approximately, 1 g of each sample was solved in 80% ethanol (20 mL) and heated in a water bath at 60°C for 1 h. Then, samples were centrifuged at 6100 rpm for 5 min, and the supernatant was passed through the filter paper and concentrated at room temperature for 16 h. About 750 μL of extracted samples were mixed with 3 mL of glycolic acid/sulfuric acid in ratio of 1:1. The absorbance of samples were read at 527 nm after 30 min and the total saponin content was expressed as mg/100 g of oleanolic acid equivalent per 100 g dry sample (Saeid et al. 2023).

### 2.6 Phytic Acid Content

About 0.5 ± 0.05 g of powdered samples were mixed with hydrochloric acid (10 mL, 0.64 N) and kept at the shaker at 300 rpm for 16 h. After 16 h, samples were centrifuged at 3000 rpm at 10°C for 20 min. Then, NaCl (1.0 ± 0.05 g) was added to filtrated supernatant and shaken at 300 rpm for 20 min. The samples were kept at −20°C for 20 min and centrifuged at 3000 rpm for 20 min. The volume of 1 mL of the supernatant was separated and diluted by deionized distilled water (25 mL). Thereafter, 1 mL of the wade reagent (0.03% FeCl_3_∙6H_2_O + 0.3% sulfosalicylic acid) was added to 3 mL of diluted sample and centrifuged at 3000 rpm for 10 min. The absorbance of samples was recorded at 500 nm. The phytic acid content of the samples was expressed as gram of phytic acid per 1000 g of dry sample (Saeid et al. 2023).

### 2.7 Statistical Analysis

The data analysis was done by one‐way analysis of variance (ANOVA) using SPSS statistical software (SPSS 21.0 for Windows, SPSS Inc., Chicago, IL, USA). Statistical significance was considered at *p ≤* 0.05, and results were expressed as mean ± SD.

## 3 Results and Discussion

### 3.1 Total Phenolic Content

Phenolic compounds with antioxidant properties could reduce the risk of cardiovascular disease in which reactive oxygen species are responsible (Çelik and Gökmen 2020). The quantity of total phenolic compounds and their nature play a detrimental in antioxidant activity determination (Veenashri and Muralikrishna 2011). As shown in Table 1, TPC is found in the range of 19.22–23.99 mg GAE/100 g belong to S_1_ and control samples respectively. Generally, ACP treatment decreased the quantity of TPC in quinoa seeds compared with the control sample (*p ≤* 0.05). However, a gradual increase in TPC is found by increasing the exposure voltage (from 50 to 60 kV). The impact of exposure time on TPC is more obvious at ACP‐treated samples at higher voltage. In other words, while no significant difference was observed between S_1_ and S_2_ samples (treated at a voltage of 50 kV and 5 and 10 min respectively) a significant (*p ≤* 0.05) increase in TPC content of S_4_ (i.e., treated for 10 min at 60 kV for 10 min) is observed compared with S_3_ (i.e., treated for 10 min at 60 kV for 5 min). Therefore, the lowest reduction in phenolic content compared with control samples was reported in S_4_ sample.

**TABLE 1:** Values of measured parameters of control and treatment samples.

Treatments	Parameters				
	FRAP	Phenolic	Flavonoids	DPPH	TBARs
Control	278.96 ± 0.47^a^	23.99 ± 0.37^a^	360.57 ± 0.86^e^	94.80 ± 0.31^a^	1.40 ± 0.06^a^
S_1_	211.02 ± 0.94^c^	19.22 ± 0.31^d^	659.52 ± 0.59^b^	90.60 ± 0.84^c^	1.18 ± 0.08^b^
S_2_	228.97 ± 0.81^b^	19.29 ± 0.18^d^	580.24 ± 0.27^d^	92.84 ± 0.18^b^	1.13 ± 0.08^c^
S_3_	181.34 ± 0.28^d^	20.86 ± 0.53^c^	621.86 ± 0.92^c^	79.63 ± 0.92^e^	1.15 ± 0.06^c^
S_4_	211.96 ± 0.64^c^	21.81 ± 0.11^b^	714.93 ± 0.19^a^	82.88 ± 0.93^d^	1.07 ± 0.06^d^

*Note:* The values presented are expressed as mean ± standard deviation of triplicate experiments. Different Lowercase letters mean significant difference based on the one‐way analysis of variance; *p ≤* 0.05. S_1_: The quinoa grains exposed to plasma treatment at 50 kV for 5 min; S_2_: The quinoa grains exposed to plasma treatment at 50 kV for 10 min; S_3_: The quinoa grains exposed to plasma treatment at 60 kV for 5 min; S_4_: The quinoa grains exposed to plasma treatment at 60 kV for 10 min.

The reduction in phenolic compounds compared with the control sample can be related to reaction of hydroxyl radicals as a plasma reactive species, atomic oxygen, or singlet oxygen changes in the chemical structure. In the other words, phenols could change or oxidize during oxidation process by polyphenol oxidase or peroxidase (Scaglioni et al. 2014). The interaction of exposure time and voltage of ACP treatment on TPC content can be attributed to phenylalanine ammonia‐lyase activation which is crucial enzyme in synthesis of phenolic compounds as a defense mechanism (Sruthi et al. 2022; Ramazzina et al. 2015). Also, plasma treatment could depolymerize or destruct the cell wall polysaccharides which facilitate extraction of the conjugated phenolic compounds (Sruthi et al. 2022; Zhu et al. 2010). This increase is compatible with comparison result of S_3_ (20.86 ± 0.53 GAE/100 g) with S_1_ (19.22 ± 0.31 GAE/100 g) and S_4_ (21.81 ± 0.11 GAE/100 g). These results are in agreement with result of TPC in blueberry juice (Hou et al. 2019), pomegranate juice (Kovačević et al. 2016), and cashew apple juice (Rodríguez et al. 2017) after plasma treatment. Thermal treatment had same effect on total phenolic contents of quinoa, while cooking with high temperature reduced the total phenolic compounds (Scaglioni et al. 2014). High voltage plasma treatment had reduced the total phenolic content of white grape juice (Pankaj et al. 2017). Therefore, it can be stated that the parameters of the cold plasma treatment process are essential in the extraction of polyphenols from food. Parameters such as plasma voltage and treatment time play an important role in the final content of bioactive compounds in plasma‐treated foods (Pogorzelska‐Nowicka et al. 2021).

### 3.2 Total Flavonoid Content

The flavonoids are well known for their beneficial and activities including anticancer, antidiabetic, and antiviral activities (Mozaffarian and Wu 2018). According to Table 1, the TFC content was reported for control, S_1_, S_2_, S_3_, and S_4_ at 360.57, 659.52, 580.24, 621.86, and 714.93 mg rutin/100 g, respectively. Overall, the significant increase (*p ≤* 0.05) was found at all ACP‐treated samples (S_1_, S_2_, S_3_ and S_4_) compared with control one by increasing both the exposure time and voltage as mentioned previously by Mehta et al. (2022). So, the highest TFC is found at S_4_ sample which is treated at 60 kV for 10 min. This increment in TFC is probably ascribed to creation of free radical factors (Hussein 2022). In addition, the biosynthesis of flavonol, flavones, phenylpropanoid, and other specific metabolites of phenolic compounds could be activated by plasma treatment which increased the flavonoid content (Sruthi et al. 2022). According to Table 1, increasing the ACP treatment's exposure time and voltage a slight decrease has been observed with values equal to 580.24 ± 0.27 and 621.86 ± 0.92 mg rutin/100 g for S_2_ and S_3_ respectively. It has been reported that release and accessibility of flavonoids and phenolics bound to the cell membranes require specific energy intensity. Consequently, energy supply by ACP treatment is susceptible to improve its total content in the food matrix (Almeida et al. 2015). On the contrary some studies have reported decrease in TPC and TFC in orange juice (Alves Filho et al. 2019) and apple juice (Nasiru et al. 2021). These differences in findings are attributed to differences in food matrices (Muhammad et al. 2018).

### 3.3 Antioxidant Capacity

The antioxidant capacity of food products could be evaluated by different methods considering the varieties existed between their bioactive compounds action mechanisms and potential synergistic reactions between them (Pogorzelska‐Nowicka et al. 2021). In the current study, reduction in ferric ions (FRAP) and the scavenging capacity of DPPH radicals was used for the determination of antioxidant activity.

The DPPH assay is based on electron transfer and actually measures the capacity of an antioxidant to reduce oxidants. DPPH is a stable radical which its reduction leads to its discoloration and is related to the antioxidants concentration and their capacity to deliver hydrogen (Nisa et al. 2019; Dudonne et al. 2009). DPPH could detect the hydrophilic and lipophilic antioxidant compounds (Muhtadi and Wiyono 2021). The FRAP assay is related to electron transfer of Fe^3+^(III) to Fe^2+^ (II) which is resulted in color change from yellow and/or colorless to blue (Huang, Ou, and Prior 2005) which could determine the hydrophilic antioxidant compounds (Arjmand et al. 2023). In FRAP, the variation in the reaction time of the antioxidants with Fe^3+^ resulted in different results. Therefore, a single‐point absorption endpoint may not show a complete reaction (Munteanu and Apetrei 2021). Also, the changes in antioxidant capacity in the presence of ACP treatment could be related to the exposure time, the intensity of plasma processing, and the bioactive response of the product (Pankaj et al. 2017).

According to Table 1, DPPH radical of control, S_1_, S_2_, S_3_, and S_4_ samples were 94.80 ± 0.31, 90.60 ± 0.84, 92.84 ± 0.18, 79.63 ± 0.92, and 82.88% ± 0.93%, respectively. Also the FRAP were 278.96 ± 0.47, 211.02 ± 0.94, 228.97 ± 0.81, 181.34 ± 0.28, and 211.96 ± 0.64 mMol Fe_2_SO_4_ for control, S_1_, S_2_, S_3_, and S_4_, respectively. However, a general decrease in antioxidant activity is found by ACP treatment in both FRAP and DPPH analysis, a significant increase is observed by increasing the exposure time of ACP treatment at constant voltage (by considering S_2_ and S_4_ compared with S_1_ and S_3_, respectively). In general, the antioxidant capacity of quinoa seeds has been decreased significantly as a result of plasma treatment compared with the control sample (*p ≤* 0.05). Also, it can be seen that increasing the exposure time of ACP treatment at a constant exposure voltage of 50 and 60 kV in S_2_ and S_4_ samples compared with S_1_ and S_3_ ones has increased the antioxidant capacity (FRAP and DPPH). However, a decrease in antioxidant capacity (FRAP, DPPH) was found by increasing the exposure voltage of ACP treatment at a constant time of 5 and 10 min. In general, it has been found that ACP treatment of samples at 50 kV for 10 min (S_2_ samples) has preserved the antioxidant capacity more effectively compared with other samples.

In the present study, the reduction in antioxidant capacity by DPPH and FRAP could be related to the reduction in total phenol content induced by reactive oxygen species, the reaction of phenol compounds, and the reduction in ascorbic acid (Sruthi et al. 2022).

The increase observed at antioxidant activity by increasing the ACP treatment time is similar with Zhang et al., who indicated an increasing trend in antioxidant capacity by passing time which is probably due to etching at structure induced by reactive species of ACP treatment which facilitate the process of antioxidants release (Zhang et al. 2019).

Considering the impact of ACP treatment on the antioxidant capacity despite reports existed about the reduction in antioxidant activity after ACP treatments in apple juice (Liao et al. 2018), blueberry juice (Hou et al. 2019), and fresh‐cut cucumbers (Wang et al. 2012), no significant changes in the antioxidant capacity of kiwifruits (Ramazzina et al. 2015), and onion powder (Kim et al. 2017) is also observed. Therefore, some parameters including plasma generation source, type of food products, mode of exposure, and treatment are considered as important parameters in this regard (Pankaj, Wan, and Keener 2018).

Other studies also showed that low and limited exposure to plasma increased the antioxidant activity, whereas exposure for extended times at elevated flow rates directed a drop in the antioxidant activity. Therefore, it is likely, it was ascertained that the intensity of plasma processing, exposure time, and types of active species plays an influential role in the retention of antioxidant components after plasma processing, and moderate plasma treatment can well maintain the antioxidant activity of the treated sample (Sruthi et al. 2022).

### 3.4 TBARs Assay

Lipid oxidation is a major concern for foods, which could lead to undesirable changes in the color, taste, odor, and shelf‐life. Lipid oxidation is a complex process involving free radical chain mechanisms forming fatty acyl peroxides or other oxidation products (Pankaj, Wan, and Keener 2018). The thiobarbituric acid reactive substance (TBARs) assay is used to measure antioxidant activities and lipid peroxidation in food products and chemical reactions, Since CP is often considered as an advanced oxidation process, it is essential to analyze its influence on the lipids present in foods. However, based on the reported studies, treatment time and plasma gas could be considered as critical factors affecting lipid oxidation (Pankaj, Wan, and Keener 2018).

As shown in Table 1, there is significant decrease in TBARs of all ACP‐treated samples (S_1_, S_2_, S_3_, and S_4_) compared with control sample. This reduction might be related to decrease in the activity of the lipase after cold plasma treatment which is resulted in the inhibition of lipid oxidation (Sutar et al. 2021). The most probable cause of enzyme inactivation was attributed to the formation of active species in plasma and their reaction with the protein structure. The reaction of free radicals with enzyme can cause changes in protein structure and mainly secondary structure in terms of loss of alpha‐helical structure and modification of some amino acids of side chains of enzyme (Tolouie et al. 2018). As a result of inactivation of the lipase enzyme, lipid peroxidation also decreases. In line with the current results, Bahrami et al. indicated a significant decrease in free fatty acid and phospholipid of wheat flour by increasing the time and voltage of cold plasma treatment (Bahrami et al. 2016).

### 3.5 Saponin Content

Saponin contains steroidal or triterpene glycosides present in heterogeneous group and is basically attached to glycosyl bonds at C‐3 and C‐17 (through C‐28) points via covalent bound, which are found in a wide variety of plants used by humans, including quinoa (Jaddu et al. 2024). Saponins as a natural compound found in quinoa seeds with the range of 0.1–5 g/100 g (Karovičová et al. 2020) is bitter in taste and limited its widespread consumption (Ruales and Nair 1993).

**FIGURE 1:**
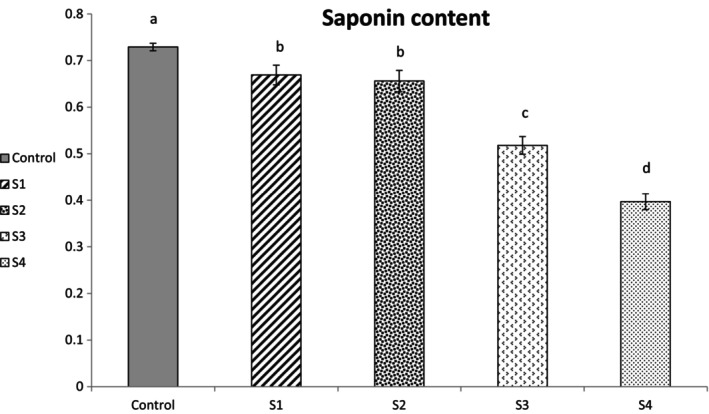
Saponin content of control and treatment samples. The values presented are expressed as mean ± standard deviation of triplicate experiments. Different Lowercase letters mean significant difference based on the one‐way analysis of variance; *p ≤* 0.05. S_1_: The quinoa grains exposed to plasma treatment at 50 kV for 5 min; S_2_: The quinoa grains exposed to plasma treatment at 50 kV for 10 min; S_3_: The quinoa grains exposed to plasma treatment at 60 kV for 5 min; S_4_: The quinoa grains exposed to plasma treatment at 60 kV for 10 min.

**FIGURE 2:**
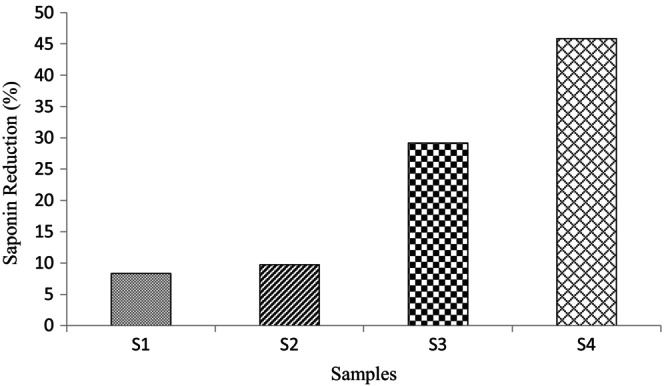
Saponin reduction in treatment samples in comparison with control. S_1_: The quinoa grains exposed to plasma treatment at 50 kV for 5 min; S_2_: The quinoa grains exposed to plasma treatment at 50 kV for 10 min; S_3_: The quinoa grains exposed to plasma treatment at 60 kV for 5 min; S_4_: The quinoa grains exposed to plasma treatment at 60 kV for 10 min.

Saponin content of control, S_1_, S_2_, S_3_, and S_4_ samples were recorded as 0.72 ± 0.00, 0.66 ± 0.02, 0.65 ± 0.02, 0.51 ± 0.01, and 0.39 ± 0.01 mg/100 g, respectively, as depicted at Figure 1. As can be seen, by increasing the voltage and exposure time of ACP treatment, the saponin content was decreased (*p ≤* 0.05). Among the ACP‐treated samples, more significant decrease has been found at S_4_ (i.e., 60 KV at 10 min) which is equal to 45.83. This reduction is about 8.33%, 9.72% and 29.16% for S_1_, S_2_, and S_3_, respectively (as depicted as Figure 2). Saponins are known as phytochemical compounds with at least one glycosidic bond between their aglycone and sugar chain (El Aziz, Ashour, and Melad 2019). Warne et al. (2021) have reported polymerization of carbohydrates mostly through glycosylation by ACP treatment. During plasma treatment, the breakage of glycosidic bonds through their interaction with the reactive oxygen species (ROS) could occur (Warne et al. 2021) which could be resulted in breakdown of the saponin structure. The lowest amount observed at S_4_ is probably due to the increase in voltage and treatment time, because it has been reported that with the increase in the time and voltage of cold plasma treatment, more glycosidic bonds are broken and the concentration of reducing sugars increases (Carvalho et al. 2021; Warne et al. 2021).

### 3.6 Phytic Acid Content

The phosphorus in seeds could be stored as myo‐inositol hexaphosphate or phytic acid (PA). The metal ions chelating activity of PA which could reduce the bioavailability of important micronutrients, have been proven (Perera, Seneweera, and Hirotsu 2018). Phytic acid could be found in the outer layers and endosperm of quinoa with concentration ranged from 200 to 880 mg/100 g^1^ (Pathan and Siddiqui 2022).

**FIGURE 3:**
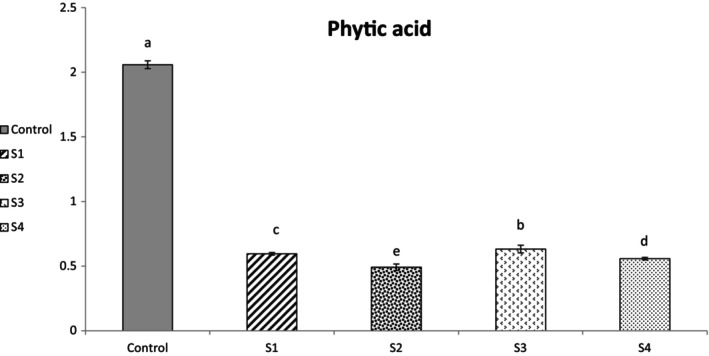
Phytic acid content of control and treatment samples. The values presented are expressed as mean ± standard deviation of triplicate experiments. Different Lowercase letters mean significant difference based on the one‐way analysis of variance; *p* ≤ 0.05. S_1_: The quinoa grains exposed to plasma treatment at 50 kV for 5 min; S_2_: The quinoa grains exposed to plasma treatment at 50 kV for 10 min; S_3_: The quinoa grains exposed to plasma treatment at 60 kV for 5 min; S_4_: The quinoa grains exposed to plasma treatment at 60 kV for 10 min.

The phytic acid content in the samples in the present study was 2.05, 0.59, 0.49, and 0.63 and 0.55 g/1000 g for control, S_1_, S_2_, S_3_, and S_4_, respectively (Figure 3). Results indicated a significant decrease in phytic acid content of quinoa compared with control sample (*p ≤* 0.05). Increasing the exposure time of ACP treatment at constant voltages of 50 and 60 kV significantly decreased the phytic acid content and the highest decrease was observed at phytic acid content of S_2_. During ACP treatment, ionized gas contained highly excited ionic and radicals could produce (Sruthi et al. 2022) that free radicals could decrease the phytic acid content by splitting the phytate ring (Sarkar et al. 2023). Our results are in agreement of El‐Niely (2007) findings, who showed the efficiency of irradiation in reduction of phytic acid in cereal grains and legumes (El‐Niely 2007). Also it has been reported that CP treatment can improve the activity of enzymes such as phytase through processing of certain food systems (Sruthi et al. 2022). Also, the decrease in phytic acid content may be related to the higher activity of phytase enzyme. Enzymes have a protein structure and the contact time and treatment voltage has an important effect on their structure. In fact, with increasing voltage intensity and constant treatment time, the enzyme activity decrease (Tang et al. 2022). On the other hand, the contact time is directly related to the phytase enzyme activity, so that it has been reported that the activity of the phytase enzyme increases with the increase ACP treatment time (Farasat et al. 2018), so the maximum observation of phytate reduction in S_2_ is probably due to the same reasons (low voltage vs. maximum exposure time).

The reduction in phytic acid content through processing is appealing as it improves the mineral bioavailability of final product (Sadhu et al. 2017). Considering the study conducted by Sadhu et al. (2017), the increased activity of phytase enzyme in mung beans has been found at quantities equal to 52% by, ACP treatment at 60 W for 20 min (Sadhu et al. 2017).

## 4 Conclusions

The potential of ACP treatment to reduce antinutritional compounds and maintain nutritional value (antioxidant capacity) of quinoa grains has been investigated. The significant impact of ACP on antinutritional factors (phytic acid and saponin) of quinoa seeds has been approved. As demonstrated all ACP‐treated samples show significantly lower quantity of saponin and phytic acid compared with the untreated sample ACP treatment at 60 kV for 10 min and 50 kV for 10 min (i.e., S_4_ and S_2_) have been found as the most effective ones in reduction of saponin and phytic acid, respectively. Considering the antioxidant capacity of ACP‐treated samples (DPPH and FRAP), the lowest decrease is observed at S_2_ which is treated at 50 kV for 10 min. Significant decrease in and increase in flavonoid content of ACP‐treated samples were also found compared with the control sample which is more obvious at S_4_ sample. Therefore, cold plasma treatment at 60 KV for 10 min could be used to reduce antinutritional compounds and as well as maintain the antioxidant compounds of quinoa grains.

## Author Contributions

**Sanaz Arjmand:** conceptualization (equal), investigation (equal), methodology (equal), project administration (equal), resources (equal), software (equal), writing – original draft (equal). **Elham Khalili Sadrabad:** conceptualization (equal), formal analysis (equal), investigation (equal), project administration (equal), software (equal), supervision (equal), visualization (equal), writing – review and editing (equal). **Fereshteh Ramroudi:** conceptualization (equal), formal analysis (equal), methodology (equal), software (equal), validation (equal), visualization (equal), writing – review and editing (equal). **Neda Mollakhalili‐meybodi:** conceptualization (equal), data curation (equal), formal analysis (equal), funding acquisition (equal), investigation (equal), methodology (equal), project administration (equal), resources (equal), software (equal), supervision (equal), validation (equal), visualization (equal), writing – review and editing (equal).

## Conflicts of Interest

The authors declare no conflicts of interest.

## Data Availability

The data are available from the corresponding author upon reasonable request.

## References

[fsn34691-bib-0001] Almeida, F. D. L. , R. S. Cavalcante , P. J. Cullen , et al. 2015. “Effects of Atmospheric Cold Plasma and Ozone on Prebiotic Orange Juice.” Innovative Food Science & Emerging Technologies 32: 127–135.

[fsn34691-bib-0002] Alves Filho, E. G. , T. H. S. Rodrigues , F. A. N. Fernandes , et al. 2019. “An Untargeted Chemometric Evaluation of Plasma and Ozone Processing Effect on Volatile Compounds in Orange Juice.” Innovative Food Science & Emerging Technologies 53: 63–69.

[fsn34691-bib-0003] Amiri, M. , M. Arab , E. Khalili Sadrabad , N. Mollakhalili‐Meybodi , and H. Fallahzadeh . 2023. “Effect of Gamma Irradiation Treatment on the Antioxidant Activity, Phenolic Compounds and Flavonoid Content of Common Buckwheat.” Radiation Physics and Chemistry 212: 111127.

[fsn34691-bib-0004] Arjmand, S. , N. Mollakhalili‐Meybodi , F. Akrami Mohajeri , F. Madadizadeh , and E. Khalili Sadrabad . 2023. “Quinoa Dough Fermentation by Saccharomyces Cerevisiae and Lactic Acid Bacteria: Changes in Saponin, Phytic Acid Content, and Antioxidant Capacity.” Food Science & Nutrition 11: 7594–7604. 38107108 10.1002/fsn3.3679 PMC10724584

[fsn34691-bib-0005] Bahrami, N. , D. Bayliss , G. Chope , S. Penson , T. Perehinec , and I. D. Fisk . 2016. “Cold Plasma: A New Technology to Modify Wheat Flour Functionality.” Food Chemistry 202: 247–253. 26920291 10.1016/j.foodchem.2016.01.113 PMC4778607

[fsn34691-bib-0006] Baier, M. , J. Foerster , U. Schnabel , et al. 2013. “Direct Non‐thermal Plasma Treatment for the Sanitation of Fresh Corn Salad Leaves: Evaluation of Physical and Physiological Effects and Antimicrobial Efficacy.” Postharvest Biology and Technology 84: 81–87.

[fsn34691-bib-0007] Carvalho, A. P. M. G. , D. R. Barros , L. S. da Silva , et al. 2021. “Dielectric Barrier Atmospheric Cold Plasma Applied to the Modification of Ariá (*Goeppertia Allouia*) Starch: Effect of Plasma Generation Voltage.” International Journal of Biological Macromolecules 182: 1618–1627. 34052266 10.1016/j.ijbiomac.2021.05.165

[fsn34691-bib-0008] Çelik, E. E. , and V. Gökmen . 2020. “Effects of Fermentation and Heat Treatments on Bound‐Ferulic Acid Content and Total Antioxidant Capacity of Bread Crust‐Like Systems Made of Different Whole Grain Flours.” Journal of Cereal Science 93: 102978.

[fsn34691-bib-0009] Danesi, F. , and A. Bordoni . 2008. “Effect of Home Freezing and Italian Style of Cooking on Antioxidant Activity of Edible Vegetables.” Journal of Food Science 73, no. 6: H109–H112. 19241586 10.1111/j.1750-3841.2008.00826.x

[fsn34691-bib-0010] Dini, I. , G. C. Tenore , and A. Dini . 2010. “Antioxidant Compound Contents and Antioxidant Activity Before and After Cooking in Sweet and Bitter *Chenopodium quinoa* Seeds.” LWT‐ Food Science and Technology 43, no. 3: 447–451.

[fsn34691-bib-0011] Dudonne, S. , X. Vitrac , P. Coutiere , M. Woillez , and J.‐M. Mérillon . 2009. “Comparative Study of Antioxidant Properties and Total Phenolic Content of 30 Plant Extracts of Industrial Interest Using DPPH, ABTS, FRAP, SOD, and ORAC Assays.” Journal of Agricultural and Food Chemistry 57, no. 5: 1768–1774. 19199445 10.1021/jf803011r

[fsn34691-bib-0012] El Aziz, M. , A. Ashour , and A. Melad . 2019. “A Review on Saponins From Medicinal Plants: Chemistry, Isolation, and Determination.” Journal of Nanomedical Research 8, no. 1: 282–288.

[fsn34691-bib-0013] El Hazzam, K. , J. Hafsa , M. Sobeh , et al. 2020. “An Insight Into Saponins From Quinoa ( *Chenopodium quinoa* Willd): A Review.” Molecules 25, no. 5: 1059. 32120971 10.3390/molecules25051059 PMC7179108

[fsn34691-bib-0014] El‐Niely, H. F. 2007. “Effect of Radiation Processing on Antinutrients, In‐Vitro Protein Digestibility and Protein Efficiency Ratio Bioassay of Legume Seeds.” Radiation Physics and Chemistry 76, no. 6: 1050–1057.

[fsn34691-bib-0015] Farasat, M. , S. Arjmand , S. O. Ranaei Siadat , Y. Sefidbakht , and H. Ghomi . 2018. “The Effect of Non‐Thermal Atmospheric Plasma on the Production and Activity of Recombinant Phytase Enzyme.” Scientific Reports 8, no. 1: 16647. 30413721 10.1038/s41598-018-34239-4 PMC6226467

[fsn34691-bib-0016] Ghasemi, R. , F. Akrami Mohajeri , A. heydari , S. A. Yasini , A. Dehghani Tafti , and E. Khalili Sadrabad . 2023. “Application of Pomegranate Peel Extract, a Waste Agricultural Product, as a Natural Preservative in Tahini.” International Journal of Food Science 2023: 8860476.

[fsn34691-bib-0017] Golmohamadi, A. , G. Möller , J. Powers , and C. Nindo . 2013. “Effect of Ultrasound Frequency on Antioxidant Activity, Total Phenolic and Anthocyanin Content of Red Raspberry Puree.” Ultrasonics Sonochemistry 20, no. 5: 1316–1323. 23507361 10.1016/j.ultsonch.2013.01.020

[fsn34691-bib-0018] Gupta, K. , P. Yadav , P. M. Dikkar , M. Sharma , and B. Singh . n.d. Advances in Food Science .

[fsn34691-bib-0019] Han, Z. , R. Shi , and D.‐W. Sun . 2020. “Effects of Novel Physical Processing Techniques on the Multi‐Structures of Starch.” Trends in Food Science and Technology 97: 126–135.

[fsn34691-bib-0020] Hou, Y. , R. Wang , Z. Gan , et al. 2019. “Effect of Cold Plasma on Blueberry Juice Quality.” Food Chemistry 290: 79–86. 31000059 10.1016/j.foodchem.2019.03.123

[fsn34691-bib-0021] Huang, D. , B. Ou , and R. L. Prior . 2005. “The Chemistry Behind Antioxidant Capacity Assays.” Journal of Agricultural and Food Chemistry 53, no. 6: 1841–1856. 15769103 10.1021/jf030723c

[fsn34691-bib-0022] Hussein, H.‐A. A. 2022. “Influence of Radio‐Grain Priming on Growth, Antioxidant Capacity, and Yield of Barley Plants.” Biotechnology Reports 34: e00724. 35686017 10.1016/j.btre.2022.e00724 PMC9171454

[fsn34691-bib-0023] Jaddu, S. , S. Sonkar , D. Seth , et al. 2024. “Cold Plasma: Unveiling Its Impact on Hydration, Rheology, Nutritional, and Anti‐Nutritional Properties in Food Materials–An Overview.” Food Chemistry: X 22: 101266. 38486618 10.1016/j.fochx.2024.101266 PMC10937106

[fsn34691-bib-0024] James, L. E. A. 2009. “Quinoa ( *Chenopodium quinoa* Willd.): Composition, Chemistry, Nutritional, and Functional Properties.” Advances in Food and Nutrition Research 58: 1–31. 19878856 10.1016/S1043-4526(09)58001-1

[fsn34691-bib-0025] Jeong, S.‐M. , S.‐Y. Kim , D.‐R. Kim , et al. 2004. “Effect of Heat Treatment on the Antioxidant Activity of Extracts From Citrus Peels.” Journal of Agricultural and Food Chemistry 52, no. 11: 3389–3393. 15161203 10.1021/jf049899k

[fsn34691-bib-0026] Karovičová, J. , Z. Kohajdová , L. Minarovičová , et al. 2020. “Utilisation of Quinoa for Development of Fermented Beverages.” Potravinarstvo 14, no. 1: 465–472.

[fsn34691-bib-0027] Keenan, D. F. , N. P. Brunton , T. R. Gormley , F. Butler , B. K. Tiwari , and A. Patras . 2010. “Effect of Thermal and High Hydrostatic Pressure Processing on Antioxidant Activity and Colour of Fruit Smoothies.” Innovative Food Science & Emerging Technologies 11, no. 4: 551–556.

[fsn34691-bib-0028] Kheto, A. , A. Mallik , R. Sehrawat , K. Gul , and W. Routray . 2023. “Atmospheric Cold Plasma Induced Nutritional & Anti‐Nutritional, Molecular Modifications and In‐Vitro Protein Digestibility of Guar Seed ( *Cyamopsis tetragonoloba* L.) Flour.” Foodservice Research International 168: 112790. 10.1016/j.foodres.2023.112790 37120236

[fsn34691-bib-0029] Kim, J. E. , Y. J. Oh , M. Y. Won , K.‐S. Lee , and S. C. Min . 2017. “Microbial Decontamination of Onion Powder Using Microwave‐Powered Cold Plasma Treatments.” Food Microbiology 62: 112–123. 27889137 10.1016/j.fm.2016.10.006

[fsn34691-bib-0030] Kovačević, D. B. , P. Putnik , V. Dragović‐Uzelac , S. Pedisić , A. R. Jambrak , and Z. Herceg . 2016. “Effects of Cold Atmospheric Gas Phase Plasma on Anthocyanins and Color in Pomegranate Juice.” Food Chemistry 190: 317–323. 26212976 10.1016/j.foodchem.2015.05.099

[fsn34691-bib-0031] Koziol, M. J. 1991. “Afrosimetric Estimation of Threshold Saponin Concentration for Bitterness in Quinoa ( *Chenopodium quinoa* Willd).” Journal of the Science of Food and Agriculture 54, no. 2: 211–219.

[fsn34691-bib-0032] Liao, X. , J. Li , A. I. Muhammad , et al. 2018. “Application of a Dielectric Barrier Discharge Atmospheric Cold Plasma (DBD‐ACP) for *Eshcerichia coli* Inactivation in Apple Juice.” Journal of Food Science 83, no. 2: 401–408. 29355961 10.1111/1750-3841.14045

[fsn34691-bib-0033] Lim, J. G. , H. M. Park , and K. S. Yoon . 2020. “Analysis of Saponin Composition and Comparison of the Antioxidant Activity of Various Parts of the Quinoa Plant ( *Chenopodium quinoa* Willd.).” Food Science & Nutrition 8, no. 1: 694–702. 31993193 10.1002/fsn3.1358 PMC6977472

[fsn34691-bib-0034] Mehta, D. , K. Yadav , K. Chaturvedi , U. S. Shivhare , and S. K. Yadav . 2022. “Impact of Cold Plasma on Extraction of Polyphenol From De‐Oiled Rice and Corn Bran: Improvement in Extraction Efficiency, in Vitro Digestibility, Antioxidant Activity, Cytotoxicity and Anti‐Inflammatory Responses.” Food and Bioprocess Technology 15, no. 5: 1142–1156.

[fsn34691-bib-0035] Miranda, M. , A. Vega‐Gálvez , J. López , et al. 2010. “Impact of Air‐Drying Temperature on Nutritional Properties, Total Phenolic Content and Antioxidant Capacity of Quinoa Seeds ( *Chenopodium quinoa* Willd.).” Industrial Crops and Products 32, no. 3: 258–263.

[fsn34691-bib-0036] Misra, N. , S. Kaur , B. K. Tiwari , A. Kaur , N. Singh , and P. Cullen . 2015. “Atmospheric Pressure Cold Plasma (ACP) Treatment of Wheat Flour.” Food Hydrocolloids 44: 115–121.

[fsn34691-bib-0037] Mozaffarian, D. , and J. H. Y. Wu . 2018. “Flavonoids, Dairy Foods, and Cardiovascular and Metabolic Health.” Circulation Research 122, no. 2: 369–384. 29348256 10.1161/CIRCRESAHA.117.309008 PMC5781235

[fsn34691-bib-0038] Muhammad, A. I. , X. Liao , P. J. Cullen , et al. 2018. “Effects of Nonthermal Plasma Technology on Functional Food Components.” Comprehensive Reviews in Food Science and Food Safety 17, no. 5: 1379–1394. 33350151 10.1111/1541-4337.12379

[fsn34691-bib-0039] Muhtadi, M. , and A. A. F. Wiyono . 2021. “Testing Antioxidant Activity of *Plumeria alba* and *Plumeria rubra* Ethanolic Extracts Using DPPH and Frap Methods and Determining Their Total Flavonoid and Phenolic Levels.” Journal of Nutraceuticals and Herbal Medicine 3, no. 2: 38–50.

[fsn34691-bib-0040] Munteanu, I. G. , and C. Apetrei . 2021. “Analytical Methods Used in Determining Antioxidant Activity: A Review.” International Journal of Molecular Sciences 22, no. 7: 3380. 33806141 10.3390/ijms22073380 PMC8037236

[fsn34691-bib-0041] Nasiru, M. M. , E. B. Frimpong , U. Muhammad , et al. 2021. “Dielectric Barrier Discharge Cold Atmospheric Plasma: Influence of Processing Parameters on Microbial Inactivation in Meat and Meat Products.” Comprehensive Reviews in Food Science and Food Safety 20, no. 3: 2626–2659. 33876887 10.1111/1541-4337.12740

[fsn34691-bib-0042] Nisa, K. , V. Rosyida , S. Nurhayati , A. Indrianingsih , C. Darsih , and W. Apriyana . 2019. “Total Phenolic Contents and Antioxidant Activity of Rice Bran Fermented With Lactic Acid Bacteria.” In IOP Conference Series: Earth and Environmental Science. Tangerang, Indonesia: IOP Publishing.

[fsn34691-bib-0043] Pankaj, S. K. , Z. Wan , W. Colonna , and K. M. Keener . 2017. “Effect of High Voltage Atmospheric Cold Plasma on White Grape Juice Quality.” Journal of the Science of Food and Agriculture 97, no. 12: 4016–4021. 28195339 10.1002/jsfa.8268

[fsn34691-bib-0044] Pankaj, S. K. , Z. Wan , and K. M. Keener . 2018. “Effects of Cold Plasma on Food Quality: A Review.” Food 7, no. 1: 4. 10.3390/foods7010004 PMC5789267 29301243

[fsn34691-bib-0045] Pathan, S. , and R. A. Siddiqui . 2022. “Nutritional Composition and Bioactive Components in Quinoa ( *Chenopodium quinoa* Willd.) Greens: A Review.” Nutrients 14: 558. 35276913 10.3390/nu14030558 PMC8840215

[fsn34691-bib-0046] Patras, A. , N. Brunton , S. Da Pieve , F. Butler , and G. Downey . 2009. “Effect of Thermal and High Pressure Processing on Antioxidant Activity and Instrumental Colour of Tomato and Carrot Purées.” Innovative Food Science & Emerging Technologies 10, no. 1: 16–22.

[fsn34691-bib-0047] Perera, I. , S. Seneweera , and N. Hirotsu . 2018. “Manipulating the Phytic Acid Content of Rice Grain Toward Improving Micronutrient Bioavailability.” Rice 11: 1–13. 29327163 10.1186/s12284-018-0200-y PMC5764899

[fsn34691-bib-0048] Pogorzelska‐Nowicka, E. , M. M. Hanula , M. Brodowska‐Trębacz , et al. 2021. “The Effect of Cold Plasma Pretreatment on Water‐Suspended Herbs Measured in the Content of Bioactive Compounds, Antioxidant Activity, Volatile Compounds and Microbial Count of Final Extracts.” Antioxidants 10, no. 11: 1740. 34829611 10.3390/antiox10111740 PMC8615236

[fsn34691-bib-0049] Ramazzina, I. , A. Berardinelli , F. Rizzi , et al. 2015. “Effect of Cold Plasma Treatment on Physico‐Chemical Parameters and Antioxidant Activity of Minimally Processed Kiwifruit.” Postharvest Biology and Technology 107: 55–65.

[fsn34691-bib-0050] Rodríguez, Ó. , W. F. Gomes , S. Rodrigues , and F. A. Fernandes . 2017. “Effect of Indirect Cold Plasma Treatment on Cashew Apple Juice ( *Anacardium occidentale* L.).” LWT 84: 457–463.

[fsn34691-bib-0051] Ruales, J. , and B. M. Nair . 1993. “Saponins, Phytic Acid, Tannins and Protease Inhibitors in Quinoa ( *Chenopodium quinoa* , Willd) Seeds.” Food Chemistry 48, no. 2: 137–143.

[fsn34691-bib-0052] Sadhu, S. , R. Thirumdas , R. Deshmukh , and U. Annapure . 2017. “Influence of Cold Plasma on the Enzymatic Activity in Germinating Mung Beans (*Vigna radiate*).” LWT 78: 97–104.

[fsn34691-bib-0053] Saeid, S. , N. Mollakhalili‐meybodi , F. A. Mohajeri , F. Madadizadeh , and E. K. Sadrabad . 2023. “The Effect of Gamma Irradiation Treatment on Quinoa Flour: Quantification of Saponin, Phytic Acid, Antioxidant Activity, and Oxidative Properties.” Radiation Physics and Chemistry 216: 111429.

[fsn34691-bib-0054] Sarkar, A. , T. Niranjan , G. Patel , A. Kheto , B. K. Tiwari , and M. Dwivedi . 2023. “Impact of Cold Plasma Treatment on Nutritional, Antinutritional, Functional, Thermal, Rheological, and Structural Properties of Pearl Millet Flour.” Journal of Food Process Engineering 46, no. 5: e14317.

[fsn34691-bib-0055] Scaglioni, P. T. , T. D. de Souza , C. G. Schmidt , and E. Badiale‐Furlong . 2014. “Availability of Free and Bound Phenolic Compounds in Rice After Hydrothermal Treatment.” Journal of Cereal Science 60, no. 3: 526–532.

[fsn34691-bib-0056] Sruthi, N. , K. Josna , R. Pandiselvam , A. Kothakota , M. Gavahian , and A. M. Khaneghah . 2022. “Impacts of Cold Plasma Treatment on Physicochemical, Functional, Bioactive, Textural, and Sensory Attributes of Food: A Comprehensive Review.” Food Chemistry 368: 130809. 34450498 10.1016/j.foodchem.2021.130809

[fsn34691-bib-0057] Suárez‐Estrella, D. , L. Torri , M. A. Pagani , and A. Marti . 2018. “Quinoa Bitterness: Causes and Solutions for Improving Product Acceptability.” Journal of the Science of Food and Agriculture 98, no. 11: 4033–4041. 29485194 10.1002/jsfa.8980

[fsn34691-bib-0058] Sutar, S. A. , R. Thirumdas , B. B. Chaudhari , R. R. Deshmukh , and U. S. Annapure . 2021. “Effect of Cold Plasma on Insect Infestation and Keeping Quality of Stored Wheat Flour.” Journal of Stored Products Research 92: 101774.

[fsn34691-bib-0059] Tang, L. , S. Hatab , J. Yan , et al. 2022. “Changes in Biochemical Properties and Activity of Trypsin‐Like Protease ( *Litopenaeus vannamei* ) Treated by Atmospheric Cold Plasma (ACP).” Food 11, no. 9: 1277. 10.3390/foods11091277 PMC9105110 35564000

[fsn34691-bib-0060] Tolouie, H. , M. A. Mohammadifar , H. Ghomi , A. S. Yaghoubi , and M. Hashemi . 2018. “The Impact of Atmospheric Cold Plasma Treatment on Inactivation of Lipase and Lipoxygenase of Wheat Germs.” Innovative Food Science & Emerging Technologies 47: 346–352.

[fsn34691-bib-0061] Veenashri, B. , and G. Muralikrishna . 2011. “In Vitro Anti‐Oxidant Activity of Xylo‐Oligosaccharides Derived From Cereal and Millet Brans–A Comparative Study.” Food Chemistry 126, no. 3: 1475–1481.

[fsn34691-bib-0062] Vilcacundo, R. , and B. Hernández‐Ledesma . 2017. “Nutritional and Biological Value of Quinoa ( *Chenopodium quinoa* Willd.).” Current Opinion in Food Science 14: 1–6.

[fsn34691-bib-0063] Wang, R. , W. Nian , H. Wu , et al. 2012. “Atmospheric‐Pressure Cold Plasma Treatment of Contaminated Fresh Fruit and Vegetable Slices: Inactivation and Physiochemical Properties Evaluation.” European Physical Journal D 66: 1–7.

[fsn34691-bib-0064] Warne, G. R. , P. M. Williams , H. Q. Pho , N. N. Tran , V. Hessel , and I. D. Fisk . 2021. “Impact of Cold Plasma on the Biomolecules and Organoleptic Properties of Foods: A Review.” Journal of Food Science 86, no. 9: 3762–3777. 34337748 10.1111/1750-3841.15856

[fsn34691-bib-0065] Wie, H. J. , H. L. Zhao , J. H. Chang , Y. S. Kim , I. K. Hwang , and G. E. Ji . 2007. “Enzymatic Modification of Saponins From *Platycodon grandiflorum* With *Aspergillus niger* .” Journal of Agricultural and Food Chemistry 55, no. 22: 8908–8913. 17927135 10.1021/jf0716937

[fsn34691-bib-0066] Xiang, Q. , L. Huangfu , S. Dong , et al. 2023. “Feasibility of Atmospheric Cold Plasma for the Elimination of Food Hazards: Recent Advances and Future Trends.” Critical Reviews in Food Science and Nutrition 63, no. 20: 4431–4449. 34761962 10.1080/10408398.2021.2002257

[fsn34691-bib-0067] Yael, B. , G. Liel , B. Hana , H. Ran , and G. Shmuel . 2012. “Total Phenolic Content and Antioxidant Activity of Red and Yellow Quinoa ( *Chenopodium quinoa* Willd.) Seeds as Affected by Baking and Cooking Conditions.” Food and Nutrition Sciences 2012: 1150–1155.

[fsn34691-bib-0068] Zahoranová, A. , L. Hoppanová , J. Šimončicová , et al. 2018. “Effect of Cold Atmospheric Pressure Plasma on Maize Seeds: Enhancement of Seedlings Growth and Surface Microorganisms Inactivation.” Plasma Chemistry and Plasma Processing 38, no. 5: 969–988.

[fsn34691-bib-0069] Zare, L. , N. Mollakhalili‐Meybodi , H. Fallahzadeh , and M. Arab . 2022. “Effect of Atmospheric Pressure Cold Plasma (ACP) Treatment on the Technological Characteristics of Quinoa Flour.” LWT 155: 112898.

[fsn34691-bib-0070] Zhang, X.‐L. , C.‐S. Zhong , A. S. Mujumdar , et al. 2019. “Cold Plasma Pretreatment Enhances Drying Kinetics and Quality Attributes of Chili Pepper ( *Capsicum annuum* L.).” Journal of Food Engineering 241: 51–57.

[fsn34691-bib-0071] Zhu, F. , Y.‐Z. Cai , J. Bao , and H. Corke . 2010. “Effect of γ‐Irradiation on Phenolic Compounds in Rice Grain.” Food Chemistry 120, no. 1: 74–77.

